# Supplementary material for: Application of Quantum—Markov Open System Models to Human Cognition and Decision
Source: Entropy (Basel). 2020 Sep 4;22(9):990. doi: 10.3390/e22090990 (PMC7597313; doi:10.3390/e22090990)
Supplement: Supplementary file 1 [file entropy-22-00990-s001.pdf]

If the density is at equilibrium so that $\frac{d}{dt}\rho = 0$, then

$$-i(1-\alpha)(H\rho - \rho H) + \alpha \begin{bmatrix} \sum_n \rho_{nn} K_{1,n} & & & \\ & \sum_n \rho_{nn} K_{2,n} & & \\ & & \ddots & \\ & & & \sum_n \rho_{nn} K_{\mathrm{N},n} \end{bmatrix} = 0$$

$$\alpha \begin{bmatrix} \sum_n \rho_{nn} K_{1,n} & & & \\ & \sum_n \rho_{nn} K_{2,n} & & \\ & & \ddots & \\ & & & \sum_n \rho_{nn} K_{\mathrm{N},n} \end{bmatrix}$$

$$= i(1-\alpha) \begin{bmatrix} \sum_n H_{1,n}\rho_{n,1} & \sum_n H_{1,n}\rho_{n,2} & \cdots & \sum_n H_{1,n}\rho_{n,N} \\ \sum_n H_{2,n}\rho_{n,1} & \sum_n H_{2,n}\rho_{n,2} & \cdots & \sum_n H_{2,n}\rho_{n,N} \\ \vdots & \vdots & \ddots & \vdots \\ \sum_n H_{N,n}\rho_{n,1} & \sum_n H_{\mathrm{N},n}\rho_{n,2} & \cdots & \sum_n H_{\mathrm{N},n}\rho_{n,\mathrm{N}} \end{bmatrix}$$

$$-i(1-\alpha) \begin{bmatrix} \sum_n \rho_{1,n}H_{n,1} & \sum_n \rho_{1,n}H_{n,2} & \cdots & \sum_n \rho_{1,n}H_{n,N} \\ \sum_n \rho_{2,n}H_{n,1} & \sum_n \rho_{2,n}H_{n,2} & \cdots & \sum_n \rho_{2,n}H_{n,N} \\ \vdots & \vdots & \ddots & \vdots \\ \sum_n \rho_{N,n}H_{n,1} & \sum_n \rho_{\mathrm{N},n}H_{n,2} & \cdots & \sum_n \rho_{\mathrm{N},n}H_{n,\mathrm{N}} \end{bmatrix}$$

This is matrix equation. Every entry should equal.

Diagonal: $\alpha \sum_n \rho_{nn} K_{j,n} = i(1-\alpha)\left(\sum_n H_{\mathrm{j},n}\rho_{n,\mathrm{j}} - \sum_n \rho_{\mathrm{j},n} H_{n,\mathrm{j}} \right)$

$$= i(1-\alpha)\left(H_{\mathrm{j,j}}\rho_{\mathrm{j,j}} + \sum_{n\neq j} H_{\mathrm{j},n}\rho_{n,\mathrm{j}} - \sum_{n\neq j} \rho_{\mathrm{j},n} H_{n,\mathrm{j}} - \rho_{\mathrm{j,j}} H_{\mathrm{j,j}} \right)$$

$$= i(1-\alpha)\left(\sum_{n\neq j} H_{\mathrm{j},n}\rho_{n,\mathrm{j}} - \sum_{n\neq j} \rho_{\mathrm{j},n} H_{n,\mathrm{j}} \right)$$

$$= i(1-\alpha)\left(\sum_{n\neq j} H_{\mathrm{j},n}\rho_{n,\mathrm{j}} - \sum_{n\neq j} \bar{\rho}_{n,\mathrm{j}} \bar{H}_{\mathrm{j},n} \right)$$

$$=i(1-\alpha)\left(\sum_{n\neq j} H_{\mathrm{j},n}\rho_{n,\mathrm{j}} - \sum_{n\neq j}\bar{H}_{\mathrm{j},n}\bar{\rho}_{n,\mathrm{j}}\right)$$
$$=i(1-\alpha)\sum_{n\neq j}\left(H_{\mathrm{j},n}\rho_{n,\mathrm{j}} - \bar{H}_{\mathrm{j},n}\bar{\rho}_{n,\mathrm{j}}\right)$$
$$=i(1-\alpha)\sum_{n\neq j} 2i\,\mathrm{Im}\left(H_{\mathrm{j},n}\rho_{n,\mathrm{j}}\right)$$
$$=-2(1-\alpha)\sum_{n\neq j}\mathrm{Im}\left(H_{\mathrm{j},n}\rho_{n,\mathrm{j}}\right)$$

Nondiagonal: $0=\sum_n H_{i,n}\rho_{n,\mathrm{j}} - \sum_n \rho_{\mathrm{i},n}H_{n,\mathrm{j}}$

$$=H_{i,i}\rho_{i,\mathrm{j}} + H_{i,j}\rho_{\mathrm{j,j}} + \sum_{n\neq i,j} H_{i,n}\rho_{n,\mathrm{j}} - \sum_{n\neq i,j}\rho_{\mathrm{i},n}H_{n,\mathrm{j}} - \rho_{\mathrm{i,i}}H_{\mathrm{i,j}} - \rho_{\mathrm{i,j}}H_{\mathrm{j,j}}$$

$$\Rightarrow H_{i,j}\left(\rho_{\mathrm{i,i}} - \rho_{\mathrm{j,j}}\right) = \left(H_{i,i} - H_{\mathrm{j,j}}\right)\rho_{i,\mathrm{j}} + \sum_{n\neq i,j} H_{i,n}\rho_{n,\mathrm{j}} - \sum_{n\neq i,j}\rho_{\mathrm{i},n}H_{n,\mathrm{j}}$$

There are totally NxN equations and NxN unknowns. Due to the symmetry, we only need to consider 1+2+…+N-1=(N-1)N/2 nondiagonal unknowns.

Now let both K and H be real and tri-diagonal.

Diagonal: $\alpha\sum_n \rho_{nn}K_{1,n} = -2(1-\alpha)\mathrm{Im}\left(H_{1,2}\rho_{2,1}\right)$

$$\alpha\sum_n \rho_{nn}K_{j,n} = -2(1-\alpha)\mathrm{Im}\left(H_{\mathrm{j},j-1}\rho_{j-1,\mathrm{j}} + H_{\mathrm{j},j+1}\rho_{j+1,\mathrm{j}}\right), j=2,...,N-1$$

$$\alpha\sum_n \rho_{nn}K_{\mathrm{N},n} = -2(1-\alpha)\mathrm{Im}\left(H_{\mathrm{N},N-1}\rho_{N-1,\mathrm{N}}\right)$$

Nondiagonal: $H_{i,j}\left(\rho_{\mathrm{i,i}} - \rho_{\mathrm{j,j}}\right) = \left(H_{i,i} - H_{\mathrm{j,j}}\right)\rho_{i,\mathrm{j}} + \sum_{n\neq i,j} H_{i,n}\rho_{n,\mathrm{j}} - \sum_{n\neq i,j}\rho_{\mathrm{i},n}H_{n,\mathrm{j}}$

Due to nondiagonal: $i\neq j$

Due to tri-diagonal: $H_{i,j}\neq 0$ when j = i-1 or j = i+1. $H_{i,j}=0$ when j < i-1 or j > i+1.

When j = i-1: $H_{i,i-1}\left(\rho_{\mathrm{i,i}} - \rho_{i-1,i-1}\right) = \left(H_{i,i} - H_{i-1,i-1}\right)\rho_{i,i-1} + H_{i,i+1}\rho_{i+1,i-1} - \rho_{\mathrm{i},i-2}H_{i-2,i-1}$

When j = i+1: $H_{i,i+1}\left(\rho_{\mathrm{i,i}} - \rho_{i+1,i+1}\right) = \left(H_{i,i} - H_{i+1,i+1}\right)\rho_{i,i+1} + H_{i,i-1}\rho_{i-1,i+1} - \rho_{\mathrm{i},i+2}H_{i+2,i+1}$

When j < i-1 or j > i+1:

$$0=\left(H_{i,i} - H_{\mathrm{j,j}}\right)\rho_{i,\mathrm{j}} + H_{i,i-1}\rho_{i-1,\mathrm{j}} + H_{i,i+1}\rho_{i+1,\mathrm{j}} - \rho_{\mathrm{i},j-1}H_{j-1,\mathrm{j}} - \rho_{\mathrm{i},j+1}H_{j+1,\mathrm{j}}$$

Due to symmetry of rho and H, we only consider rho_ij and H_ij with j>=i.

Diagonal: $\alpha\sum_n \rho_{nn}K_{1,n} = -2(1-\alpha)\mathrm{Im}\left(H_{1,2}\bar{\rho}_{1,2}\right)$

$$\alpha\sum_n \rho_{nn}K_{j,n} = -2(1-\alpha)\mathrm{Im}\left(\bar{H}_{j-1,j}\rho_{j-1,\mathrm{j}} + H_{\mathrm{j},j+1}\bar{\rho}_{\mathrm{j},j+1}\right), j=2,...,N-1$$

$$\alpha\sum_n \rho_{nn}K_{\mathrm{N},n} = -2(1-\alpha)\mathrm{Im}\left(\bar{H}_{N-1,\mathrm{N}}\rho_{N-1,\mathrm{N}}\right)$$

When j = i+1: $H_{i,i+1}\left(\rho_{\mathrm{i,i}} - \rho_{i+1,i+1}\right) = \left(H_{i,i} - H_{i+1,i+1}\right)\rho_{i,i+1} + \bar{H}_{i-1,i}\rho_{i-1,i+1} - \rho_{\mathrm{i},i+2}\bar{H}_{i+1,i+2}$

When j > i+1: $0=\left(H_{i,i} - H_{\mathrm{j,j}}\right)\rho_{i,\mathrm{j}} + \bar{H}_{i-1,i}\rho_{i-1,\mathrm{j}} + H_{i,i+1}\rho_{i+1,\mathrm{j}} - H_{j-1,\mathrm{j}}\rho_{\mathrm{i},j-1} - \bar{H}_{j,j+1}\rho_{\mathrm{i},j+1}$
